# Comparison of radiographic changes in the alveolar crest after extraction of fully and partially erupted premolars during orthodontic treatment: A retrospective analytical study

**DOI:** 10.34172/joddd.2021.046

**Published:** 2021-12-05

**Authors:** Sanam Darban Hosseini, Mojgan Kachoei, Masoumeh Faramarzi, Mahdiyeh Esmaeilzadeh

**Affiliations:** ^1^Private Practice, Tabriz, Iran; ^2^Department of Orthodontics, Faculty of Dentistry, Tabriz University of Medical Sciences, Tabriz, Iran; ^3^Department of Periodontics, Faculty of Dentistry, Tabriz University of Medical Sciences, Tabriz, Iran; ^4^Dental Student, Student Research Committee, Tabriz University of Medical Science, Tabriz, Iran

**Keywords:** Alveolar process, Orthodontic tooth movements, Premolar, Tooth extraction

## Abstract

**Background.** The alveolar process plays an essential role in providing dental support and gradually disappears with tooth loss. Space deficiency can cause one premolar to remain semi-erupted adjacent to a fully-erupted premolar. During orthodontic treatment, each of these premolars can be extracted. This retrospective study aimed to compare radiographic changes of the alveolar crest due to orthodontic movements of fully-erupted and semi-erupted premolars into the extraction sites before and after treatment.

**Methods.** The patients were divided into the fully-erupted premolar extraction (first) group and the semi-erupted premolar extraction (second) group. The distance between the cementoenamel junction (CEJ) and the alveolar crest, from the distal aspect of the canine to the mesial aspect of the first molar, was measured on panoramic radiographs of 78 patients (39 from each group) before and after treatment with a digital caliper. Changes in the alveolar crest were compared between the two groups. Finally, the height differences of the alveolar crest in mesial and distal aspects of the remaining premolars in both groups were calculated at the end of treatment. Descriptive statistical analyses and paired and independent *t* tests were used in the study.

**Results.** The distance from the CEJ to the alveolar crest at mesial and distal aspects in the first group and the distal aspect of the extraction site in the second group increased significantly. However, changes at the mesial aspect were not significant in the second group. Comparing the alveolar crest height between the two groups and between the mesial and distal aspects of the remaining premolar tooth indicated no significant differences.

**Conclusion.** No significant difference was observed between the extraction of a fully-erupted or semi-erupted premolar to obtain greater alveolar height.

## Introduction

The alveolar process is a part of the maxilla and mandible that forms the tooth sockets.^1^ Therefore, the alveolar bone has a critical role in providing support for teeth, and a decrease in its height leads to various problems, including tooth mobility and, finally, tooth loss. The main etiologic factors for tooth mobility include alveolar bone loss, inflammatory changes in the periodontal ligament, and occlusal trauma. Tooth mobility resulting from trauma and inflammation can be treated; however, mobility due to alveolar bone loss cannot.^1^ The progressive loss of the alveolar bone occurs due to anatomic, biologic, and mechanical factors.^2^ Mechanical stimulation resulting from mastication is necessary to maintain the health of the alveolar bone.^2^ A significant decrease in occlusal forces leads to nonfunctional atrophy, increasing the distance between the cementoenamel junction (CEJ) and the alveolar crest.^1^ The alveolar crest forms around the tooth concomitant with tooth eruption and is lost gradually with tooth loss.^1^ Therefore, the alveolar crest height decreases in the tooth extraction site, resulting in an osseous defect in that area, which in turn leads to periodontal problems.^1^ If another tooth is moved to that area with orthodontic forces, the alveolar crest height increases to some extent.^1^

Generally, tooth movement is a complicated process that involves changes in the gingiva, periodontal ligament, cementum, and alveolar bone. The effects of tooth movement on the periodontium depend on the magnitude of the applied force and its direction and duration.^3^ Teeth and their supporting structures can adapt to an individual’s functional needs during her/his life and move in the alveolar bone in a process called physiological migration of teeth. The physiologic movement of a tooth results from the normal functions of tooth-supporting tissues, and during the movement, teeth move the supra-alveolar fibers, too, resulting in the remodeling of the periodontal ligament and alveolar bone. As mentioned previously, significant changes occur in tooth positions without orthodontic movement, too. There are no differences between the tissue responses to the physical tooth movement and orthodontic movement; however, since teeth move faster during orthodontic movement, the outcomes of orthodontic forces are more extensive and significant.^3^

Many studies have evaluated the effects of orthodontic treatment and orthodontic movement of teeth on the periodontal status.^4-6^ Orthodontic treatment sometimes involves tooth extraction to create the necessary space to align teeth and move the adjacent teeth to that area. Studies have shown the effects of these treatments on the general health of the periodontium.^4-7^ Also, based on a study by Rivera Circuns and Tulloch, moving the adjacent teeth to the extracted tooth site, especially if the tooth was extracted a long time ago, might cause periodontal complications, including gingival invagination.^7^ It should be noted that moving a tooth to the area of a newly extracted tooth is associated with fewer complications due to the larger number of cells differentiating into osseous tissue, more limited amount of bone resorption, and a higher rate of bone remodeling compared to moving a tooth to an area which has been edentulous for a long time.^3^ As mentioned previously, the alveolar bone height is affected by orthodontic treatments.^8,9^ Different techniques can be used to determine the height of the alveolar bone, including clinical probing, and different radiographic techniques and CBCT as paraclinical methods.^10-15^ Gedik et al^16^ showed that among the panoramic, bitewing, and periapical radiographs, bitewing is the most accurate technique for determining the alveolar bone height, followed by panoramic, with the periapical radiograph exhibiting the lowest accuracy among them.

Several researchers have determined the height of the alveolar bone on radiographic images, and in many cases, panoramic radiographs have been used to this end. Since panoramic radiographs are used for various purposes in dentistry, evaluating the height of the alveolar bone on these radiographs is of significance.^17-19^

One of the orthodontic patients’ problems is semi-eruption of one premolar tooth with or without buccal or lingual block-out, adjacent to a fully-erupted premolar, on one side or both sides of the dental arch due to space deficiency.

In such cases, in patients whose treatment plan involves tooth extraction, there would be two options: One option entails the extraction of the semi-erupted premolar and allowing the adjacent premolar to close the space; the second option involves the extraction of the fully-erupted premolar and preserving the semi-erupted premolar. Considering the large number of orthodontic patients with the above condition and the effects of tooth eruption, extraction, and movement on the alveolar crest height and periodontal health, it is necessary to evaluate and compare changes in the height of the alveolar crest in the groups of patients described above.

The general objective of the present study was to compare radiographic changes of the alveolar crest following orthodontic movements of fully-erupted and semi-erupted premolars into the extraction sites.

Specific objectives were:

1. Determination of radiographic changes of the alveolar crest in fully-erupted premolar extraction group (group 1)
2. Determination of radiographic changes of the alveolar crest in semi-erupted premolar extraction group (group 2)
3. Comparison of the alveolar crest height changes in both groups
4. Comparison of the alveolar crest height differences at the mesial and distal aspects of the remaining premolars in both groups at the end of orthodontic treatment

## Methods

In this retrospective analytical study, the panoramic radiographs of patients, before and after orthodontic treatment, referred to the Department of Orthodontics, Faculty of Dentistry, Tabriz University of Medical Sciences two private clinics, were used. The study included two groups; in group 1, orthodontic treatment was carried out by the extraction of fully erupted premolar teeth, and in group 2, the orthodontic treatment was carried out by the extraction of semi-erupted premolar teeth. The pilot study indicated a sample size of 31 for each group; to increase the accuracy of the study, 39 samples were included in each group, totaling 78 samples in the two groups. Similar to the studies by Wical and Swoope^17^ and Packota et al,^18^ the ratio of certain distances was used in calculations to solve the problem of different magnifications on panoramic radiographs.

### Inclusion criteria

1. Undergoing fixed orthodontic treatment with the edgewise system
2. Having a sound stone cast or high-quality photographs before and after treatment
3. Having high-quality panoramic radiographs before and after treatment
4. Presence of at least one semi-erupted premolar tooth at the beginning of the treatment due to space deficiency, resulting in buccal or lingual block-out of that tooth and not reaching the occlusal level of adjacent teeth, and having a fully-erupted premolar tooth adjacent to the semi-erupted one before treatment
5. Extraction of one of the premolars during orthodontic treatment
6. Presence of the canine and first molar teeth at the beginning and end of treatment on the study side
7. Presence of fully erupted second or third molar tooth at the beginning and at the end of treatment on the study side that had not undergone orthodontic forces

### Exclusion criteria

1. Low-quality radiographs of the study area due to the superimposition of tooth images or other reasons
2. Patient’s affliction with periodontal problems before or during treatment
3. Rotation of the teeth to be studied (canine, premolars, and first molar)
4. Complete block-out of the semi-erupted premolar, causing the mesial and distal teeth to the blocked-out one to contact each other before treatment
5. Apically positioned crown or restoration in one of the teeth to be evaluated during the orthodontic treatment
6. Overhanging of the restoration margin (which can cause alveolar bone loss)

### Evaluation of panoramic images

The radiographs were evaluated under standard displaying conditions in a dimly lit room. A digital caliper with 0.01 mm accuracy was used to measure the shortest distance between the CEJ and the most occlusal point of the alveolar crest on the panoramic radiographs of patients with a treatment plan involving extraction of the fully erupted premolar tooth (group 1) or semi-erupted premolar tooth (group 2). The measurements were made at the distal aspect of the canine tooth, the mesial and distal aspects of the first and second premolar teeth, and the mesial aspect of the first molar tooth before treatment. The same measurements were carried out at the distal aspect of the canine tooth, the mesial and distal aspects of the first or second (remaining) premolar tooth, and the mesial aspect of the first molar tooth at the end of treatment. All the measurements were taken three times, twice by one observer (the researcher’s assistant) with a one-week interval and once by the second observer (the researcher) to increase the accuracy. The means of the measurements were reported as the distance between the CEJ and the alveolar crest. When the CEJ was above the crest, the numeric values were reported positive; otherwise, they were reported negative. Finally, the difference between the distances measured before and after treatment was calculated.

Besides, to eliminate the effect of different magnifications of panoramic radiographs, relative magnification of the panoramic radiographs before and after treatment was calculated. To fulfill this aim, the size of fully-erupted second or third molar teeth – not subjected to orthodontic forces – was determined on the before panoramic views, from the summit of a specific cusp to the apex of a specific root in the quadrant under study and called “a.” Then, the measurement of the size of the same tooth was repeated on the panoramic view after treatment and called “b.” The ratio of “a” to “b” (a/b) was multiplied by the distance between the CEJ and the alveolar crest at the end of treatment, and the resulting numeric value was used in calculations.

To evaluate the radiographic changes of the alveolar crest in both groups, if the first premolar was extracted, distal of the canine and mesial of the second premolar were reported as the mesial and distal of the extraction site, respectively, and if the second premolar tooth was extracted, distal of the first premolar and mesial of the first molar were reported as the mesial and distal of the extraction site respectively, and the results were compared in each study group before and after the treatment, and between both groups.

Finally, the distance from the CEJ to the alveolar crest on the side next to the extraction area of the remaining premolar was subtracted from the distance from the CEJ to the alveolar crest on the other side of the same tooth at the end of the treatment to compare the differences of the alveolar crest height on both sides of the remaining premolars in both exaction groups.

### Data analysis

Descriptive statistics were used to determine the mean distances between the CEJ and the alveolar crest before and after orthodontic treatment, and the differences were analyzed with paired *t* test. SPSS 16 was used for analyses, and statistical significance was set at *P* < 0.05.

## Results

Seventy-nine patients were evaluated in the present study, consisting of 52 females and 27 males. The first premolar was extracted in 58 patients, and the second premolar was extracted in 21 patients. Of all the patients, 18, 33, 19, 6, and 3 patients were under orthodontic treatment for 1, 2, 3, 4, and 5 years, respectively.

### Intraclass and interclass examiner errors

Cronbach’s alpha coefficient for the scores calculated from the first and second observers’ observations showed the reliability of these scores.

Tables 1 and 2 present the mean distances between the CEJ and the alveolar crest before and after treatment in patients whose fully erupted first or second premolars were extracted.

**Table 1:** Comparison of radiographic changes of the alveolar crest in the group in which a fully erupted first premolar was extracted

**Tooth aspects**	**N**	**Mean±SD**	***P*** value*
The distal aspect of canine - Before	23	0.65 ± 0.88	0.00
The distal aspect of canine - After	23	1.08 ± 0.93	
The mesial aspect of 2nd premolar - Before	29	-0.07 ± 1.43	0.02
The mesial aspect of 2nd premolar - After	29	0.65 ± 0.81	
The distal aspect of 2nd premolar - Before	29	0.24 ± 0.70	0.04
The distal aspect of 2nd premolar - After	29	0.51 ± 0.63	
The mesial aspect of 1st molar -Before	29	1.09 ± 0.74	0.10
The mesial aspect of 1st molar -After	29	0.85 ± 0.95	

*P < 0.05 is statistically significant.

**Table 2:** Comparison of radiographic changes of the alveolar crest in the group in which a fully erupted second premolar was extracted

**Tooth aspects**	**N**	**Mean±SD**	***P*** value*
The distal aspect of canine - Before	8	0.50 ± 1.35	0.98
The distal aspect of canine- After	8	0.49 ± 1.05	
The mesial aspect of 1st premolar - Before	10	-0.28 ± 1.06	0.02
The mesial aspect of 1st premolar - After	10	0.57 ± 0.51	
The distal aspect of 1st premolar - Before	10	-0.55 ± 1.32	0.13
The distal aspect of 1st premolar - After	10	0.23 ± 0.72	
The mesial aspect of 1st molar - Before	10	0.47 ± 0.37	0.02
The mesial aspect of 1st molar - after	10	0.74 ± 0.41	

*P < 0.05 is statistically significant.

Comparison of radiographic changes of the alveolar crest in the group in which fully erupted teeth were extracted indicated that the mean distances between the CEJ and the alveolar crest in the mesial aspect of the extraction site before and after treatment were 0.29 and 0.82 mm, respectively. Furthermore, in the distal aspect of the extraction site, the distances were 0.07 and 0.68 mm, respectively, before and after treatment. The increase was significant on both sides (*P* < 0.05).

Tables 3 and 4 present the numeric values of the mean distances between the CEJ and the alveolar crest before and after treatment in patients whose semi-erupted teeth (first or second premolars) were extracted.

**Table 3:** Comparison of radiographic changes of the alveolar crest in the group in which a semi-erupted first premolar was extracted

**Tooth aspects**	**N**	**Mean±SD**	***P*** value*
The distal aspect of canine - Before	22	0.91 ± 0.82	0.21
The distal aspect of canine - After	22	1.15 ± 0.41	
The mesial aspect of 2nd premolar - Before	29	0.40 ± 0.59	0.00
The mesial aspect of 2nd premolar - After	29	0.960.43	
The distal aspect of 2nd premolar - Before	29	0.520.62	0.57
The distal aspect of 2nd premolar - After	29	0.580.51	
The mesial aspect of 1st molar - Before	29	0.610.41	0.01
The mesial aspect of 1st molar - After	29	0.860.38	

*P < 0.05 is statistically significant.

**Table 4:** Comparison of radiographic changes of the alveolar crest in the group in which a semi-erupted second premolar was extracted

**Tooth aspects**	**N**	**Mean±SD**	***P*** value*
The distal aspect of canine - Before	7	0.80 ± 0.92	0.97
The distal aspect of canine - After	7	0.81 ± 0.56	
The mesial aspect of 1st premolar - Before	11	0.38 ± 0.70	0.10
The mesial aspect of 1st premolar - After	11	0.86 ± 0.59	
The distal aspect of 1st premolar - Before	11	0.95 ± 1.68	0.91
The distal aspect of 1st premolar - After	11	0.91 ± 0.74	
The mesial aspect of 1st molar - Before	11	0.94 ± 0.65	0.52
The mesial aspect of 1st molar - After	11	0.84 ± 0.72	

^*^ *P* < 0.05 is statistically significant

The mean distances between the CEJ and the alveolar crest in the mesial aspect of the extraction site in groups in which semi-erupted teeth were extracted before and after treatment were 0.93 and 1.07 mm, respectively, with no significant differences (*P* > 0.05). However, the mean distances on the distal aspect of the extraction site in the groups mentioned above before and after treatment were 0.55 and 0.93 mm, indicating a significant increase after treatment (*P* < 0.05).

According to Table 5, changes in the mean distance of CEJ to the alveolar crest at the mesial aspect of the extraction site in the first and second group were 0.53 and 0.14 mm, respectively, and at the distal aspect, the changes in the first and second group were 0.60 and 0.37 mm, respectively. The differences between the two groups on both sides were not statistically significant. Moreover, the mean differences of the distance of CEJ to the alveolar crest between the mesial and distal of the remaining premolar teeth at the end of treatment in the first and second group were 0.12 and 0.15 mm, respectively, with no statistically significant differences between the two groups.

**Table 5:** Comparison of radiographic changes in the alveolar crest in the two groups with the extraction of fully erupted and semi-erupted premolar teeth

**Tooth aspect**	**Fully erupted premolar teeth extraction**	**Semi-erupted premolar teeth extraction**	***P*** ** value** ^*^
	**Mean±SD**	**Mean±SD**	
The mesial aspect of the extraction site	0.14 ± 0.95	0.53 ± 0.90	0.09
The distal aspect of the extraction site	0.37 ± 0.67	0.66 ± 1.45	0.36

^*^ *P* < 0.05 is statistically significant

## Discussion

A semi-erupted premolar tooth with or without buccal or lingual block-out in one or both quadrants adjacent to a fully-erupted premolar is one of the manifestations of tooth crowding and one of the conditions that indicates the need for orthodontic treatment.^3^

The treatment plan in such conditions usually includes the extraction of one of the premolar teeth.^3^ The present study evaluated the radiographic changes of the alveolar crest after extraction of fully-erupted premolars compared to semi-erupted premolars and the orthodontic movement of teeth into the space of the extracted tooth.

Since tooth eruption, extraction, and tooth movements affect the alveolar crest height and since the alveolar bone has a vital role in supporting teeth and the teeth-supporting structures are predominantly confined to the coronal two-thirds of the root, even a slight decrease in the height of the alveolar bone has many clinical manifestations.^3^ Therefore, evaluating the details of different treatment modalities is useful for achieving greater alveolar bone heights. In the present study, similar to the study by Kim et al,^19^ the distance between the alveolar crest and the CEJ was measured on panoramic radiographs to determine the alveolar bone height.

If bitewing radiographs had been used in the present study, similar to a study by Gedik et al,^16^ more reliable results would have possibly been achieved. However, due to the retrospective nature of the study and the unavailability of bitewing radiographs in patient files from the areas evaluated, panoramic radiographs were used.

Baxter^20^ determined the distance between the CEJ and the most occlusal part of the alveolar process from the distal aspect of the canine teeth to the mesial aspect of the second molar teeth on bitewing radiographs of 76 individuals who had undergone orthodontic treatment. Of all the 76 individuals, the treatment plan of 15 did not include tooth extraction, but the treatment plan of the rest included the extraction of the first or second premolar teeth. A general and slight decrease, i.e., <0.5 mm, in the alveolar crest height was seen in patients after orthodontic treatment. In the present study, in most of the evaluated areas, a decrease was observed in the alveolar crest height. Moving the teeth toward the extraction site in Baxter’s study did not specifically affect the distance between the alveolar crest and the CEJ. However, in the present study, in the group with the extraction of the first premolar teeth, the distal aspect of the canine teeth, which were moved toward the extraction site, exhibited a significant decrease in the alveolar crest height. The difference might be attributed to the use of specific treatment mechanics (canine sectional retraction). In Baxter’s study, tooth extraction did not significantly affect the distance between the alveolar crest and the CEJ. It appears that bone moves concomitant with tooth movement, and there is a fixed distance between the alveolar crest and the CEJ, different from the results of the present study.

Castro et al^21^ evaluated the distance between the CEJ and the alveolar crest, using CBCT images before and after orthodontic treatment without extraction, and concluded that the distance increased with orthodontic treatment, similar to the conclusions in the present study in most of the evaluated areas.

Janson et al^22^ evaluated changes of the alveolar crest after orthodontic treatment involving the first premolar extraction in three different groups and an untreated control group. The distance between the CEJ and the alveolar crest was measured from the distal aspect of the canine teeth to the mesial aspect of the first molar teeth on bitewing radiographs. The results showed an increase in the distance between the CEJ and the alveolar crest in the treated group compared to the control group, especially in the areas adjacent to the extraction site, consistent with the present study.

In the present study, when the semi-erupted second premolar tooth was retained, and it erupted next to the first molar tooth, the bone mesial to the first molar tooth did not undergo much resorption. However, when the fully erupted second premolar tooth was retained, the bone on the mesial aspect of the first molar tooth underwent significant resorption, which might be due to the simultaneous eruption of the semi-erupted second premolar tooth and growth of bone.

When the semi-erupted first premolar tooth was retained, and it erupted adjacent to the canine tooth, the bone distal to the canine tooth did not undergo much resorption. Also, when the fully erupted first premolar tooth was retained, the distal aspect of the canine tooth did not undergo significant changes, which might be due to the delayed eruption of the canine tooth, resulting in the simultaneous growth of the alveolar crest in this area compared to the mesial aspect of the first molar tooth, periodontal irritation, and destruction of the band on the first molar tooth, the less need for retraction of the anterior teeth after the extraction of the second premolar tooth, compared to the first premolar tooth, resulting in less compression on the distal aspect of the canine tooth or a measurement error due to the specific location of the canine tooth on the panoramic radiographs.

## Conclusion

In treatment plans involving extraction, when there is a semi-erupted tooth out of the dental arch and a fully-erupted tooth within the dental arch, it is possibly advisable to extract the semi-erupted tooth to achieve a higher level of the alveolar crest. However, further and more accurate studies are necessary to substantiate this conclusion.

## Authors’ Contributions

The study was planned and designed by MK., MF. SDHA conducted the clinical experiments and contributed to data acquisition. The statistical analysis and interpretation of data were carried out by SDHA and MK. ME contributed to the literature review and manuscript preparation and editing. All the authors have read and approved the final manuscript.

## Acknowledgment

The authors would like to acknowledge the Research Vice-Chancellor of Tabriz University of Medical Sciences.

## Funding

This study was financially supported by the Research Vice-Chancellor of Tabriz University of Medical Sciences.

## Conflict of Interests

The authors declare no conflict of interests.

## Ethics Approval

The study protocol was approved by the Ethics Committee of Research Council of Tabriz Dental School, Tabriz, Iran (approval number: IR.TBZMED.REC.1394.680). All the procedures of the study were performed based on the Declaration of Helsinki.
